# Inverse association of serum albumin levels with diabetic retinopathy in type 2 diabetic patients: a cross-sectional study

**DOI:** 10.1038/s41598-024-54704-7

**Published:** 2024-02-18

**Authors:** Xianhua Li, Wenqing Hao, Nailong Yang

**Affiliations:** 1 Department of Endocrinology and Metabolism, The Affiliated Hospital of Qingdao University, Qingdao, China; 2 Department of Nursing and Hospital Infection Management, The Affiliated Hospital of Qingdao University, Qingdao, China

**Keywords:** Serum albumin, Diabetic retinopathy, Type 2 diabetes mellitus, Cross-sectional study, Diabetes complications, Type 2 diabetes

## Abstract

This study aimed to explore the association between serum albumin (ALB) levels and diabetic retinopathy in patients with type 2 diabetes. In this cross-sectional study, we retrospectively collected clinical data from patients with type 2 diabetes who were admitted to the Endocrinology Department of the Affiliated Hospital of Qingdao University between January 1, 2021, and December 1, 2022. All included patients underwent measurements of serum albumin levels and screening for diabetes-related complications. The association between serum albumin levels and retinopathy was assessed using logistic regression after adjusting for potential confounders. Further, stratified analyses and curve fitting were conducted to delve deeper into the relationship. After inclusion and exclusion criteria were applied, a total of 1947 patients were analyzed. Among these, 982 were male and 965 were female. The mean serum albumin level was 39.86 ± 3.27 g/L. Diabetic retinopathy was present in 41.24% of the patients. After adjusting for potential confounders, we observed a significant inverse association between serum albumin levels and the incidence of retinopathy. Specifically, for every 10 g/L increase in albumin level, the odds of retinopathy decreased (odds ratio [OR] = 0.67; 95% confidence interval [CI] = 0.48–0.94; *P* = 0.0209).The curve fitting validated the inverse relationship between serum albumin and retinopathy without evidence of non-linearity or threshold saturation effects. Stratified analyses consistently indicated no interaction effects across subgroups. This cross-sectional study identified a significant inverse relationship between serum albumin levels and diabetic retinopathy in patients with type 2 diabetes. However, due to the cross-sectional nature of this study, further prospective studies are warranted to confirm these findings.

## Introduction

Type 2 diabetes mellitus (T2DM) is a global health issue. In 2015, an estimated 415 million people worldwide had T2DM, and this number is expected to rise to 642 million by 2040^1^. As the prevalence of diabetes increases and the lifespan of patients extends, diabetic retinopathy (DR) and its associated visual impairments are also rapidly increasing worldwide^2–4^. DR is the most common microvascular complication of T2DM and is recognized as a leading cause of blindness and visual impairment in the working-age population globally^3,5^. Although numerous studies have focused on the pathogenesis and influencing factors of DR, including epigenetic mechanisms such as DNA methylation, histone modifications, as well as miRNAs and long non-coding RNA (lnc-RNA) regulation^6–8^, the exploration of the relationship between serum albumin (ALB) and DR remains inadequate. Serum albumin (ALB) is the most abundant protein in circulation and exhibits significant antioxidant activity, effectively scavenging free radicals^9,10^. Given its physiological properties, low ALB may be an underappreciated risk factor associated with DR^11,12^.

While some studies have investigated the relationship between ALB and the pathogenesis and complications of T2DM, the conclusions drawn have been diverse and contradictory^13,14^. Therefore, this study aims to delve deeper into the association between ALB levels and DR in patients with T2DM through a cross-sectional study.

## Methods

### Research setting and population

A single-center, cross-sectional analysis was conducted in the Endocrinology Department of the Affiliated Hospital of Qingdao University between January 1, 2021, and December 1, 2022. This period was chosen based on the availability of continuous data from our hospital’s internal database. It provided a complete and unbiased set of patient records, ensuring the consistency and reliability of our research. Furthermore, during this timeframe, the epidemiological trends of diabetic retinopathy and the treatment strategies for type 2 diabetes remained stable, helping to minimize the impact of external confounding factors. We included inpatients aged 18 and above with a T2DM diagnosis. Patient data were extracted from the hospital’s electronic medical records system.

### Inclusion and exclusion criteria

Inclusion Criteria:

T2DM diagnosis based on American Diabetes Association^15^.

Comprehensive eye examination records, particularly retinal status.

Ability and willingness to provide a full set of medical and laboratory data, including ALB levels.

Signed informed consent for study participation.

Exclusion Criteria:

Diagnosis of Type 1 Diabetes or gestational diabetes.

Severe systemic or ocular diseases, such as uveitis or glaucoma.

Treatments or surgeries, e.g., intravitreal injections, within the last three months.

Incomplete or inconsistent clinical and laboratory data.

**Figure 1:**
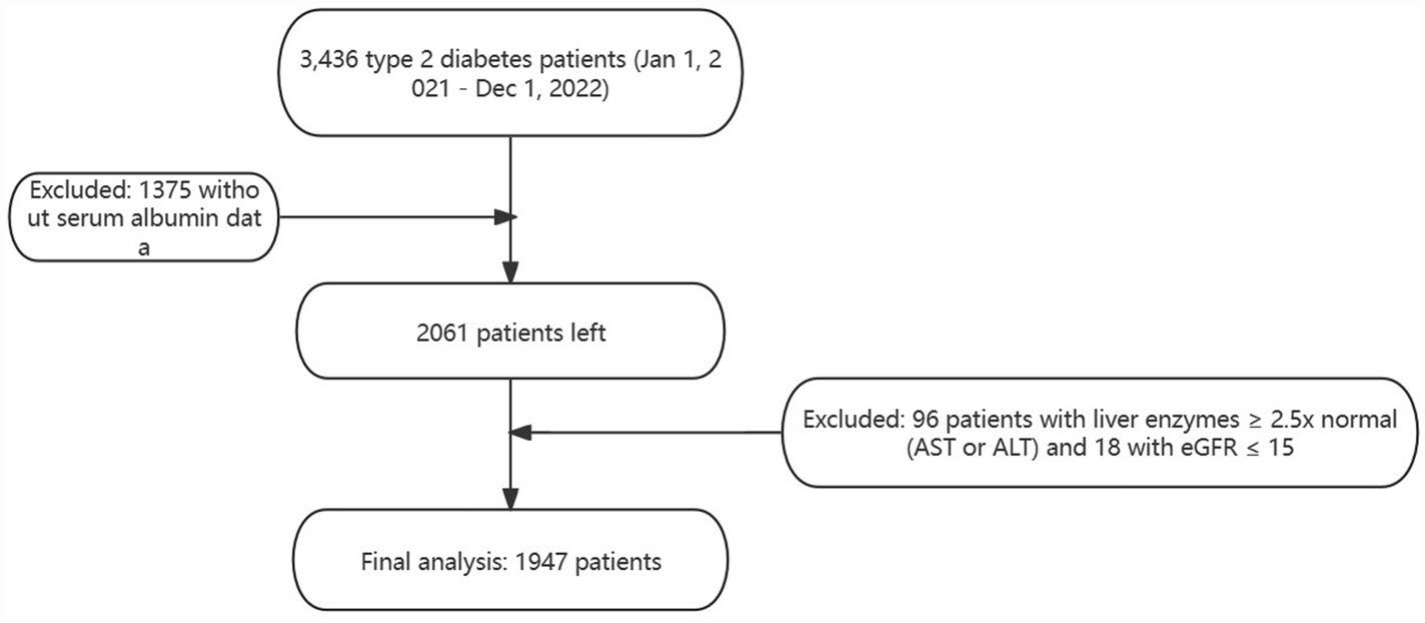
Flowchart of study population.

Participant selection is detailed in Fig. 1.

### Measurement of serum albumin

ALB levels were determined using the bromocresol green method, per manufacturer’s guidelines. The reference range is 40–55 g/L.

### DR status

DR assessment involved a slit lamp microscope and optical coherence tomography. An ophthalmologist made the DR diagnosis based on observed retinopathy changes from funduscopic examinations^16^.

### Ethics statement

This study adhered to the Declaration of Helsinki principles, procured participant informed consent, and received ethical approval from the Ethics Committee of the Affiliated Hospital of Qingdao University (QYFY WZLL 28169).

### Covariates

Demographics and Clinical Data: Information included gender, age, diabetes duration, and complications.

Anthropometrics: Data included height, weight, and BMI. BMI = weight (kg)/height^2^ (m^2^).

Lifestyle Factors: Smoking and alcohol consumption statuses were determined.

Biochemical Analyses: After 8–10 h of fasting, blood samples were collected for analysis, including lipid profiles, Cr, FPG, liver function, HbA1c, and UAER. Except for HbA1c, tests were conducted using the Hitachi 7600 analyzer. eGFR was calculated using the formula by Andrew S. Levey et al.^17^. Fatty liver was evaluated based on ultrasound. Hypertension is defined as a systolic blood pressure (SBP) of 140 mmHg or greater and/or a diastolic blood pressure (DBP) of 90 mmHg or higher, based on measurements taken in a clinical setting on three separate occasions, without the influence of antihypertensive medications^18^. Diabetic nephropathy (DKD) is characterized by patients with a documented history of type 2 diabetes mellitus (T2DM) presenting with either albuminuria (urinary albumin-to-creatinine ratio, UACR, exceeding 30 mg/g) or an estimated glomerular filtration rate (eGFR) below 60 ml/min/1.73 m^2^^19^. The diagnosis of Diabetic Peripheral Neuropathy (DPN) was established upon the analysis of clinical symptoms (such as sensory loss, pain, and muscular weakness), neurological examinations, and the outcomes of nerve conduction studies^20^.

### Statistical methods

Continuous variables were either mean [Standard Deviation (SD)] or a median [Interquartile Range (IQR)]. DR differences were evaluated using the χ^2^ test, student T-test, or Man Whitney U test. Multivariate binary logistic regression assessed the ALB-DR correlation. Nonlinearity was examined using the Generalized Additive Model. Stratified binary logistic regression was the method of choice for subgroup analyses. Statistical analyses were conducted using R software, version 4.2.0, and EmpowerStats software. *P* values < 0.05 were considered significant.

### Ethical approval

This research was conducted in accordance with the principles set forth in the Declaration of Helsinki. Informed consent was obtained from all participants. The study received the endorsement of the Ethics Committee of the Affiliated Hospital of Qingdao University (Approval No. QYFY WZLL 28169).

## Results

### Participant characterization description

In Table 1, participants were categorized based on the presence or absence of Diabetic Retinopathy (DR). Of the 1949 participants, 1144 (58.76%) did not have DR, while 803 (41.24%) did. There were significant differences between the two groups in terms of age (*P* < 0.001), duration of diabetes (*P* < 0.001), blood glucose levels (HbA1c, *P* < 0.001), and lipid levels (TG, TC, both *P* < 0.05). Furthermore, distinct differences were noted in AST (*P* < 0.001), ALT (*P* < 0.001), and the presence of comorbid conditions such as fatty liver (*P* < 0.001), diabetic nephropathy (*P* < 0.001), and diabetic peripheral neuropathy (*P* < 0.001).

**Table 1:** baseline characteristics of participants:

	Non-diabetic retinopathy (n = 1144)	Diabetic retinopathy (n = 803)	*P* value
Age (years old), mean(sd)	59.03 (12.44)	63.11 (10.33)	< 0.001
Sex, *n* (%)			0.141
Male	593 (51.84%)	389 (48.44%)	
Female	551 (48.16%)	414 (51.56%)	
BMI (kg/m^2^), n (%)			0.003
≤ 18.5	14 (1.22%)	11 (1.37%)	
> 18.5, ≤ 24	338 (29.55%)	284 (35.37%)	
> 24, ≤ 28	501 (43.79%)	356 (44.33%)	
> 28	291 (25.44%)	152 (18.93%)	
Smoking History, n (%)			0.285
Non-smokers	835 (73.25%)	604 (75.41%)	
Smokers	305 (26.75%)	197 (24.59%)	
Alcohol Consumption History, n (%)			0.968
Non-drinkers	863 (75.70%)	607 (75.78%)	
Drinkers	277 (24.30%)	194 (24.22%)	
Diabetic duration (years), median (IQR)	6.00 (2.00–10.00)	10.00 (6.00–18.00)	< 0.001
FBG (mmol/L), mean (sd)	7.23 (2.23)	7.44 (2.66)	0.062
HbA1c (%), mean (sd)	8.29 (1.98)	8.65 (1.88)	< 0.001
TG (mmol/L), median (IQR)	1.37 (0.95–2.15)	1.25 (0.89–1.85)	< 0.001
TC (mmol/L), mean (sd)	4.70 (1.17)	4.58 (1.24)	0.043
HDL-C (mmol/L), mean (sd)	1.24 (0.31)	1.26 (0.32)	0.256
LDL-C (mmol/L), mean (sd)	2.68 (0.89)	2.60 (0.95)	0.067
UA (umol/L), mean (sd)	320.55 (88.30)	313.66 (85.60)	0.087
AST(U/L), mean ± sd	19.66 (9.87)	17.95 (6.62)	< 0.001
ALT(U/L), median (IQR)	18.00 (13.00–25.00)	16.00 (12.00–22.00)	< 0.001
eGFR (mL/min per 1.73 m^2^), mean (sd)	103.80 (47.72)	103.39 (50.06)	0.855
ALB g/l, mean (sd)	40.22 (3.10)	39.35 (3.43)	< 0.001
Fatty liver disease, n (%)			< 0.001
No	531 (46.42%)	467 (58.16%)	
Yes	613 (53.58%)	336 (41.84%)	
Hypertension, n (%)			0.129
No	465 (40.65%)	299 (37.24%)	
Yes	679 (59.35%)	504 (62.76%)	
Diabetic nephropathy, n (%)			< 0.001
No	919 (80.33%)	526 (65.50%)	
Yes	225 (19.67%)	277 (34.50%)	
Diabetic peripheral neuropathy, n (%)			< 0.001
No	463 (40.47%)	134 (16.69%)	
Yes	681 (59.53%)	669 (83.31%)	

Table Results Format: (N) Mean (SD) Median (IQR)/N(%).

*P* value Calculation: For continuous variables, *P* values are derived from the Kruskal–Wallis rank-sum test. For count variables with an expected frequency of less than 10, Fisher’s exact test is employed.

FBG, Fasting Blood Glucose; HbA1c, Hemoglobin A1c; eGFR, Estimated Glomerular Filtration Rate; AST, Aspartate Aminotransferase; ALT, Alanine Aminotransferase; UA, Uric Acid; TC, Total Cholesterol; TG, Triglycerides; LDL-C, Low-Density Lipoprotein Cholesterol; HDL-C, High-Density Lipoprotein Cholesterol.

Correlation between Albumin and Diabetic Retinopathy:

Regarding serum albumin (ALB) levels presented in Table 1, the mean ALB for participants with DR was 39.35 ± 3.43 g/l, while for those without DR it was 40.22 ± 3.10 g/l. A significant association was observed between ALB levels and the presence of DR (*P* < 0.001).

As illustrated in Table 2, we evaluated the relationship between serum albumin (ALB) and diabetic retinopathy (DR) using logistic regression analysis after adjusting for potential confounders. The non-adjusted model revealed that for each 10 g/L increment in ALB, the risk of DR was significantly reduced by 56% (OR = 0.44, 95% CI 0.33, 0.58, *P* < 0.0001). This association persisted across all models. In Adjusted Model I, every 10 g/L increase in ALB was associated with a 51% decrease in DR risk (OR = 0.49, 95% CI 0.37–0.66, *P* < 0.0001). In Adjusted Model II, the corresponding decrease was 34% (OR = 0.66, 95% CI 0.48–0.94, *P* = 0.0123). In Model III, it was 33% (OR = 0.67, 95% CI 0.48–0.94, *P* = 0.0209).

**Table 2:** Relationship between Serum Albumin Levels and DR in different models.

Exposure variable	Non-adjusted model OR (95% CI) *P* value	Adjust I model OR (95% CI) *P* value	Adjust II model OR (95% CI) *P* value	Adjust III model OR (95% CI) *P* value
ALB (per 10 g/l change)	0.44 (0.33, 0.58) < 0.0001	0.49 (0.37, 0.66) < 0.0001	0.66 (0.48, 0.91) 0.0123	0.67 (0.48, 0.94) 0.0209

Non-adjusted model: No variables adjusted for.

Adjusted Model I: Adjusted for Age and Sex.

Adjusted Model II: Age; Diabetes Duration; HbA1c; ALT; EGFR; Fatty Liver Disease; Diabetic Nephropathy1.

Adjusted Model III: Adjusted for Age, Sex, BMI, Diabetes Duration, Smoking History, Fasting Blood Glucose (FBG), HbA1c, Total Cholesterol, Triglycerides, Aspartate Aminotransferase (AST), Alanine Aminotransferase (ALT), Fatty Liver Disease, Diabetic Nephropathy, and Diabetic Peripheral Neuropathy.

Image Annotation: The generalized additive model plot delineates the association between ALB levels (X-axis) and predicted DR risk (Y-axis).

**Figure 2:**
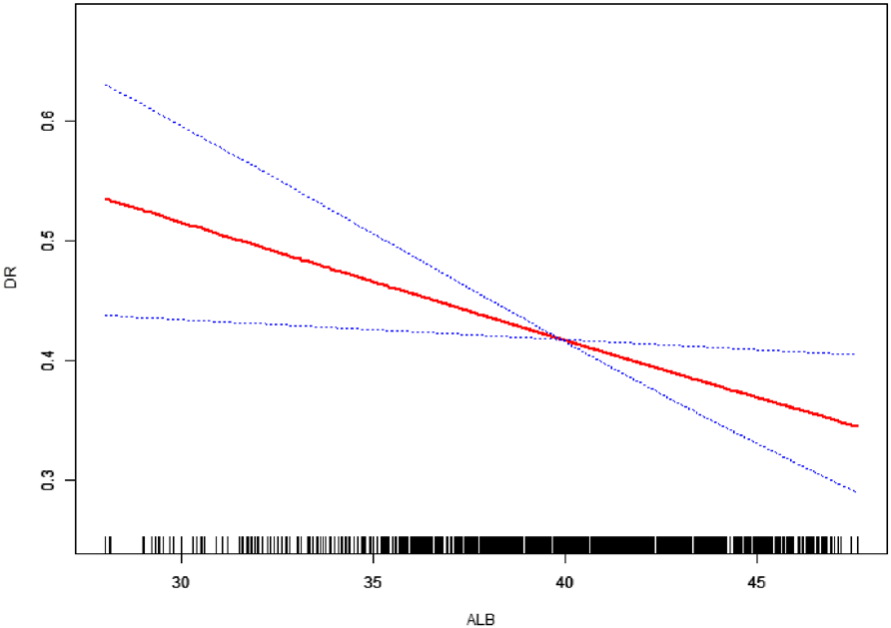
Curve Fitting: Evaluating the relationship between serum albumin levels (ALB) and diabetic retinopathy risk via generalized additive models.

To elucidate the association between ALB levels and DR risk, curve fitting analyses, as illustrated in Fig. 2, were conducted, adjusting for confounders such as age, sex, BMI, duration of diabetes, smoking status, FBG, HbA1c, lipid profile (total cholesterol and triglycerides), liver enzymes (AST and ALT), and comorbidities like fatty liver disease, diabetic nephropathy, and diabetic peripheral neuropathy. As depicted in Fig. 2, the analyses revealed a significant inverse relationship between ALB levels and DR risk. Importantly, as shown in Fig. 2, no inflection point was observed, denoting a consistent decrease in DR risk with rising ALB levels across the observed range.

As shown in Table 3, subsequent stratified analyses were conducted considering variables such as age, gender, BMI, diabetes duration, smoking status, FBG, HbA1c, fatty liver disease, diabetic nephropathy, and diabetic peripheral neuropathy. The interaction P-values for all categories were less than 0.05 in these subgroups. This consistency underscores the significant association between ALB and DR across varied conditions and demographics. Notably, the direction of association varied among some subgroups, yet the robustness of the ALB-DR relationship was unwavering, further reinforcing the pivotal role of ALB in DR pathogenesis.

**Table 3:** Stratified analyses of ALB levels and diabetic retinopathy risk.

Subgroups	Participants	OR (95% CI)	*P* for interaction
Age (years old)			0.72
Low	605	0.75 (0.38, 1.48)	
Middle	679	0.58 (0.32, 1.04)	
High	663	0.62 (0.36, 1.06)	
Sex			0.40
Male	982	0.61 (0.38, 1.00)	
Female	965	0.79 (0.49, 1.27)	
BMI (kg/m^2^)			0.07
< 24	618	0.44 (0.25, 0.79)	
24.0–28.0	872	1.05 (0.62, 1.76)	
> 28.0	457	0.53 (0.25, 1.17)	
Diabetic duration (years)			0.47
< 5.0	743	0.81 (0.43, 1.52)	
5.0–10.0	560	0.75 (0.41, 1.37)	
> 10.0	644	0.53 (0.30, 0.93)	
Smoking history			0.12
Yes	502	0.44 (0.22, 0.90)	
No	1439	0.79 (0.53, 1.16)	
FBG (mmol/L)			0.89
< 7	1020	0.68 (0.42, 1.08)	
≥ 7	909	0.70 (0.43, 1.14)	
HbA1c (%)			0.90
< 7	466	0.66 (0.30, 1.47)	
≥ 7	1376	0.66 (0.46, 0.96)	
Fatty liver disease			0.73
Yes	949	0.73 (0.43, 1.22)	
No	998	0.66 (0.42, 1.03)	
Diabetic nephropathy			1.00
Yes	502	0.68 (0.38, 1.20)	
No	1445	0.65 (0.43, 1.00	
Diabetic peripheral neuropathy			0.09
Yes	1350	0.58 (0.40, 0.85)	
No	597	1.14 (0.53, 2.43)	

## Discussion

In this cross-sectional study, we analyzed data from 1947 type 2 diabetic patients and found a significant negative correlation between serum albumin (ALB) levels and diabetic retinopathy (DR). This association remained after adjusting for major confounders such as age and gender. Although our findings are consistent with those of Gao-Xiang Wang’s 2022 study^21^, it is important to note that our study is based on data from Chinese hospitalized patients, providing further evidence for the consistency of the relationship between ALB and DR across different populations. It is worth mentioning that Wang et al. chose to conduct a quartile analysis based on ALB levels, while we opted for ALB grouping in units of 10, a method that helps reveal the strength of the association across different ranges. Furthermore, to gain a deeper understanding of this association, we performed curve fitting analysis to explore potential nonlinear relationships.

Serum albumin (ALB) is considered to have significant antioxidant activity, particularly its 34th cysteine residue, which is the most abundant source of free thiol in plasma^22,23^. This is particularly important for type 2 diabetic patients, who commonly experience oxidative stress and chronic inflammation. In these patients, the antioxidant properties of ALB may play a key role in defending against oxidative damage^24,25^.

In addition, ALB can bind to advanced glycation end products (AGEs), which are produced through non-enzymatic glycation reactions under hyperglycemic conditions ^26,27^. AGEs are closely associated with the development of diabetic retinopathy^28,29^, and ALB may mitigate their damaging effects on the retina by binding to them^30^.

Moreover, serum albumin plays a key role in maintaining vascular permeability and stability, which helps reduce vascular leakage and edema, maintaining the integrity of blood vessel walls and preventing the formation and progression of retinopathy^24,31^.

Overall, serum albumin’s antioxidant effects, binding to AGEs, and maintenance of vascular integrity provide a biological basis for its protective role in type 2 diabetic retinopathy, further explaining the negative correlation between ALB and DR observed in this study.

However, there are several key limitations in this study. Firstly, due to the cross-sectional design, we were only able to collect baseline serum albumin measurements, making it difficult to establish causation. Future studies could consider using a longitudinal cohort design to more accurately explore the relationship between serum albumin levels and diabetic retinopathy.

Secondly, although we adjusted for multiple variables, there is still the possibility of unaccounted confounding factors. Future studies could consider using matched study designs or propensity score matching to reduce the potential for confounding.

Lastly, our sample was sourced from the endocrinology department of a single hospital, which may have resulted in selection bias. To enhance the generalizability and representativeness of the study results, we recommend future research be conducted in multiple hospitals or different regions. Additionally, it’s important to note that the sample size, determined by data from 1947 patients, was not based on a priori power calculations. Nevertheless, it offered adequate statistical power (81.8%) for detecting an OR of 0.67. We acknowledge that future studies could benefit from predefined sample size estimations to enhance study design and findings. Despite these considerations, we believe the current sample size lends credibility to our results within the context of this study.

Despite these limitations, our study provides valuable insights into the relationship between serum albumin levels and type 2 diabetic retinopathy. We hope that future researchers will further explore this field, taking into account the limitations mentioned in our study.

## Conclusions

In conclusion, our research unveils a marked negative correlation between serum albumin levels and the presence of diabetic retinopathy among type 2 diabetic patients. This association holds even after accounting for confounding factors, emphasizing the potential protective role of serum albumin. Our findings not only enrich the current body of evidence but also suggest that maintaining optimal serum albumin levels could be a therapeutic target for mitigating the risk of diabetic retinopathy. As we delve deeper into this association, the insights gained hold promise for practical clinical implications, directing future research pathways and interventions in diabetes care.

## Data Availability

The data that support the findings of this study are not publicly available due to patient confidentiality concerns but are available from the corresponding authors upon reasonable request.
